# Supplementary material for: Bioactive Peptides Produced by Cyanobacteria of the Genus Nostoc: A Review
Source: Mar Drugs. 2019 Sep 29;17(10):561. doi: 10.3390/md17100561 (PMC6835634; doi:10.3390/md17100561)
Supplement: Supplementary file 1 [file marinedrugs-17-00561-s001.pdf]

Supplementary Materials

Bioactive Peptides Produced by Cyanobacteria of the Genus *Nostoc*: a Review

Anna Fidor [1], Robert Konkel [1] and Hanna Mazur-Marzec [1,2,*]

[1] Division of Marine Biotechnology, Faculty of Oceanography and Geography, University of Gdańsk, Marszałka J. Piłsudskiego 46, PL-81378 Gdynia, Poland; anna.fidor@phdstud.ug.edu.pl (A.F.); robert.konkel@phdstud.ug.edu.pl (R.K.)
[2] Institute of Oceanology, Polish Academy of Sciences, Powstańców Warszawy 55, PL-81712 Sopot, Poland
* Correspondence: biohm@ug.edu.pl; Tel.: +48-58-5236621; Fax: 48-58-5236712

Table 1. Peptides produced by cyanobacteria of the genus *Nostoc* (MW molecular weight).

Peptide name	MW	Strain, habitat & place of isolation	References
Aeruginosin 865	864	*Nostoc* sp. Lukešova 30/93	38
		Terrestrial: Forest soil, Krušné mountains, Czech Republic	
Anabaenopeptin	807	*Nostoc* sp. CENA543	117
	808	Saline/alkaline: Salina 67 Mil Lake, Brazil	
	827	*Nostoc* sp. ASN_M	30
	841	Freshwater: Paddy fields, Golestan province,	
	843	Iran	
	857	*N. punctiforme* PCC73102	115
		Terrestrial: Isolated from *Macrozamia* sp., Australia	
Banyascyclamide A	537	*Nostoc* sp. TAU IL-235	37
Banyascyclamide B	521	Freshwater: Banyas stream, Jordan River,	
Banyascyclamide C	555	Israel	
Banyaside A	992	*Nostoc* sp. TAU IL-235	123
Banyaside B	949	Freshwater: Banyas stream, Jordan River, Israel	
Banyasin A	713	*Nostoc* sp. TAU IL-235	123
		Freshwater: Banyas stream, Jordan River, Israel	
Cryptophycin-1 (A)	654	*Nostoc* sp. ATCC 53789	47
Cryptophycin-2(B)	620	Terrestrial: Lichen, Arron Island, Scotland	
Cryptophycin-3 (C)	638	*Nostoc* sp. GSV 224	36, 51
Cryptophycin-4 (D)	604	Terrestrial: India	
Cryptophycin-16	640		
Cryptophycin-17	624		
Cryptophycin-18	638		
Cryptophycin-19	624		
Cryptophycin-21	640		
Cryptophycin-23	674		
Cryptophycin-24	606		
Cryptophycin-26	638		
Cryptophycin-28	624		
Cryptophycin-29	624		
Cryptophycin-30	656		
Cryptophycin-31	688		
Cryptophycin-38	654		

Cryptophycin-40	640		
Cryptophycin-43	590		
Cryptophycin-45	658		
Cryptophycin-46	638		
Cryptophycin-49	624		
Cryptophycin-50	640		
Cryptophycin-54	654		
Cryptophycin-175	672		
Cryptophycin-176	626		
Cryptophycin-326	675		
Cryptophycin-327	655		
Cyanopeptolin 962	962	*N. edaphicum* CCNP 1411	27
Cyanopeptolin 969	969	Brackish water: Gulf of Gdańsk,	
Cyanopeptolin 978	978	Baltic Sea	
Cyanopeptolin 985	985		
Cyanopeptolin 990	990		
Cyanopeptolin 992	992		
Cyanopeptolin 999	999		
Cyanopeptolin 1006	1006		
Cyanopeptolin 1013	1013		
Cyanopeptolin 1018	1018		
Cyanopeptolin 1020	1020		
Cyanopeptolin 1027	1027		
Cyanopeptolin 1048	1048		
Cyanovirin-N	11013	*N. ellipsosporum*	24
Hassallidin 1	1295	*Nostoc* sp. CENA 219	124
Hassallidin 2	1293	Freshwater: Brazil	
Hassallidin 3	1279	*N. calcicula* 6 sf calc	125
Hassallidin 4	1279	Dobré Pole, Czech Republic	
Hassallidin 5	1277		
Hassallidin 7	1313		
Hassallidin 8	1269		
Hassallidin 10	1281		
Hassallidin 11	1297		
Hassallidin 12	1297		
Hassallidin 14	1297		
Hassallidin 15	1297		
Hassallidin 17	1263		
Hassallidin 19	1297		
Hassallidin 20	1263		
Hassallidin 22	1263		
Hassallidin 24	1265		
Hassallidin 26	1297		
Hassallidin 27	1263		
Insulapeptolide A	941	*N. insulare* SAG 54.79	113
Insulapeptolide B	955	Terrestrial: Soil	
Insulapeptolide C	955		
Insulapeptolide D	969		
Insulapeptolide E	1020		
Insulapeptolide F	1006		
Insulapeptolide G	991		
Insulapeptolide H	1005		

[MeAsp^3, Adda^5, Mdha^7] microcystin LA	908	*Nostoc* sp. BHU 001<br>Freshwater: Agricultural pond, India	126
[MeAsp^3, Adda^5, Mdha^7] microcystin AR	952	*Nostoc* sp. BHU 001<br>Freshwater: Agricultural pond, India	126
[Leu/Ile^2,Asp^3, ADDA^5, Mdha^7] microcystin	980	*Nostoc* sp. Treb K1/5<br>Pool no. 1 in the Collection of water a wetland plants, benthos Třeboň, Czech Republic	127
[MeAsp^3, DMAdda^5, Mdha^7] microcystin LR	980	*Nostoc* sp. IO-102-I<br>Terrestrial: Isolated from lichen, Sysmä, Finland<br>*Nostoc* sp. NR1<br>Freshwater: Nile River, Egypt	106<br><br><br>128
[MeAsp^3, Adda^5, Mdha^7] microcystin LR	995	*Nostoc* sp. BHU 001<br>Freshwater: Agricultural pond, India	126
[Asp^3, ADMAdda^5, Mdha^7] microcystin LR	1008	*Nostoc* sp. 152<br>Freshwater: Lake Sääskjärvi, Finland<br>*Nostoc* sp. IO-102-I<br>Terrestrial: Isolated from lichen, Sysmä, Finland	88, 103<br><br>106
[Asp^3, ADMAdda^5, Dhb^7] microcystin LR	1008	*Nostoc* sp. DUN 901<br>Brackish water: Lake, UK	104
		Nostoc sp. NR1<br>Freshwater: Nile River, Egypt	128
[MeAsp^3, DMAdda^5, Mdha^7] microcystin LR	1008	*Nostoc* sp. BHU 001<br>Freshwater: Agricultural pond, India	126
[MeAsp^3, ADMAdda^5, Mdhb^7] microcystin LR	1008	*Nostoc* sp. 152<br>Freshwater: Lake Sääskjärvi, Finland	103
[Asp^3, Adda^5, Mdha^7] microcystin FR	1014	*Nostoc* sp. Treb K1/5<br>Pool no. 1 in the Collection of water a wetland plants, benthos Třeboň, Czech Republic	127
[Asp^3, ADMAdda^5, Mdha^7] microcystin *L*-Har	1022	*Nostoc* sp. 152<br>Freshwater: Lake Sääskjärvi, Finland	103
[MeAsp^3, ADMAdda^5, Mdha^7] microcystin LR	1022	*Nostoc* sp. 152<br>Freshwater: Lake Sääskjärvi, Finland<br>*Nostoc* sp. IO-102-I<br>Terrestrial: Isolated from lichen, Sysmä, Finland	88, 103<br><br>106
[Asp^3, Adda^5, Mdha^7] microcystin YR	1030	*Nostoc* sp. Treb K1/5<br>Pool no. 1 in the Collection of water a wetland plants, benthos Třeboň, Czech Republic	127
[MeAsp^3, Adda^5, Dha^7] microcystin YR	1030	*Nostoc* sp. CENA88<br>Freshwater: Sao Paulo, Brazil	129
[MeAsp^3, ADMAdda^5, Mdha^7] microcystin *L*-Har	1036	*Nostoc* sp. 152<br>Freshwater: Lake Sääskjärvi, Finland	130
[MeAsp^3, ADMAdda^5, Mdha^7] microcystin XR	1036	*Nostoc* sp. IO-102-I<br>Terrestrial: Isolated from lichen, Sysmä, Finland	106
[Asp^3, ADMAdda^5, Dhb^7] microcystin RR	1051	*Nostoc* sp. DUN 901<br>Brackish water: Lake, UK	104
[MeAsp^3, Adda^5, Mdha^7] microcystin WR	1067	*Nostoc* sp. BHU 001<br>Freshwater: Agricultural pond, India	126

[Asp3, ADMAdda5, Dhb7] microcystin HtyR	1072	*Nostoc* sp. DUN 901<br>Brackish water: Lake, UK	104
[MeAsp3, ADMAdda5, Mdha7] microcystin XR	1076	*Nostoc* sp. IO-102-I<br>Terrestrial: Isolated from lichen, Sysmä, Finland	106
Microvirdin G<br>Microvirdin H	1805<br>1837	*N. minutum* NIES-26<br>Terrestrial: Ishigaki, Japan<br>*N. punctiforme* PCC73102<br>Terrestrial: Isolated from *Macrozamia* sp., Australia	100<br><br>95
Muscoride A	512	*N. muscorum* IAM M-14<br>Freshwater	131
Namalide B<br>Namalide D<br>Namalide E	575<br>559<br>545	*Nostoc* sp. CENA 543<br>Saline/alkaline: Salina 67 Mil Lake, Brazil	117
Namalide F	545		
Nodularin<br><br>Nodularin *L*-Har	824<br><br>838	*Nostoc* sp. 65.1<br>Terrestrial: Cycad symbiont, Australia<br>*Nostoc* sp. 73.1<br>Terrestrial: Cycad symbiont, Australia	109, 132
Nostamide A<br><br><br>Nostamide B<br>Nostamide C<br>Nostamide D<br>Nostamide E	841<br><br><br>805<br>777<br>777<br>777	*N. punctiforme* PCC73102<br>Terrestrial: Isolated from *Macrozamia* sp., Australia<br>*Nostoc* sp. CENA543<br>Saline/alkaline: Salina 67 Mil Lake, Brazil	115<br><br>117
Nostocyclamide A<br>Nostocyclamide M	490<br>506	*Nostoc* sp. 31<br>Freshwater	133, 134
Nostocyclin A (1)	1116	*Nostoc* sp. DUN 901<br>Brackish water: Lake, UK	114
Nostocyclopeptide A1<br>Nostocyclopeptide A2<br>Nostocyclopeptide A3<br><br><br><br>Nostocyclopeptide M1	756<br>790<br>804<br><br><br><br>881	*Nostoc* sp. ATCC 53789<br>Terrestrial: Isolated from lichen, Arron Island, Scotland<br>*Nostoc* sp. ASN_M<br>Freshwater: Paddy fields, Golestan province, Iran<br>*Nostoc* sp. XSPORK 13A<br>Seawater: Isolated from gastropod, Cape of Porkkala, Baltic Sea	62<br><br>30<br><br>63
Nostofungicidine	1080	*N. commune* OK-1<br>Terrestrial: Okayama, Japan	40
Nostoginin BN 741<br>Nostoginin BN 578	741<br>578	*Nostoc* sp. TAU IL-235<br>Freshwater: Banyas, Jordan River, Israel	37
Nostopeptolide A1<br>Nostopeptolide A2<br>Nostopeptolide A3<br>Nostopeptolide L1<br>Nostopeptolide L2<br>Nostopeptolide L3<br>Nostopeptolide L4	1080<br>1066<br>1080<br>1063<br>1047<br>1049<br>1033	*Nostoc* sp. GSV224<br>Terrestrial: India<br>*Nostoc* sp. XPORK 5A<br>Seawater: Isolated from gastropod, Cape of Porkkala, Baltic Sea<br>*Nostoc* sp. UK2aImI<br>Terrestrial: Isolated from lichen	93<br><br>94<br><br>94
Nostopeptin A<br>Nostopeptin B	954<br>926	*N. minutum* Nies-26<br>Terrestrial/Freshwater: Ishigaki, Japonia	112

Nostopeptin BN 920	920	*Nostoc* sp. TAU IL-235<br>Freshwater: Banyas, Jordan River, Israel	37
Nostophycin	888	*Nostoc* sp. 152<br>Freshwater: Sääksjärvi Lake, Finland	87, 92
Nostosin A<br>Nostosin B	449<br>451	*Nostoc* sp. FSN<br>Terrestrial/freshwater: Paddy fields, Golestan, Iran	97
Nostoweipeptin W1<br>Nostoweipeptin W2<br>Nostoweipeptin W3<br>Nostoweipeptin W4<br>Nostoweipeptin W5<br>Nostoweipeptin W6<br>Nostoweipeptin W7	1214<br>1198<br>1198<br>1200<br>1200<br>1200<br>1172	*Nostoc* sp. XPORK 5A<br>Brackish water: Gulf of Finland, Baltic Sea	94
Schizopeptin 791	791	*Nostoc* sp. CENA543<br>Saline/alkaline: Salina 67 Mil Lake, Brazil	117
Tenuecyclamide A<br>Tenuecyclamide B<br>Tenuecyclamide C<br>Tenuecyclamide D	459<br>459<br>505<br>521	*N. spongiaeforme* var. *tenue* TAU IL-184-6<br>Terrestrial: Soil/Litophytic sample, Bet Dagan, Israel	135

References

123. Ploutno, A.; Carmeli, S. Banyasin A and banyasides A and B, three novel modified peptides from a water bloom of the cyanobacterium *Nostoc* sp. *Tetrahedron* **2005**, *61*, 575–583.
124. Vestola, J.; Shishido, T.; Jokela, J.; Fewer, D.; Aitio, O.; Permi, P.; Wahlsten, M.; Wang, H.; Rouhiainen, L.; Sivonen, K. Hassallidins, antifungal glycolipopeptides, are widespread among cyanobacteria and are the end-product of a nonribosomal pathway. *Proc. Natl. Acad. Sci. USA* **2014**, *111*, 1909–1917.
125. Shishido, T.; Humisto, A.; Jokela, J.; Liu, L.; Wahlsten, M.; Tamrakar, A.; Fewer, D.; Permi, P.; Andreote, A.; Fiore, M.; et al. Antifungal compounds from cyanobacteria. *Mar. Drugs* **2015**, *13*, 2124–2140.
126. Bajpai, R.; Sharma, N.; Lawton, L.; Edwards, C.; Rai, A. Microcystin producing cyanobacterium *Nostoc* sp. BHU001 from a pond in India. *Toxicon* **2009**, *53*, 587–590.
127. Kust, A.; Urajová, P.; Hrouzek, P.; Vu, D.; Čapková, K.; Štenclová, L.; Řeháková, K.; Kozliková-Zapomělová, E.; Lepšová-Skácelová, O.; Lukešová, A.; et al. A new microcystin producing *Nostoc* strain discovered in broad toxicological screening of non-planktic Nostocaceae (cyanobacteria). *Toxicon* **2018**, *150*, 66–73.
128. Abu-Serie, M.; Nasser, N.; El-Wahab, A.; Shehawy, R.; Pienaar, H.; Baddour, N.; Amer, R. In vivo assessment of the hepatotoxicity of a new *Nostoc* isolate from the Nile River: *Nostoc* sp. strain NR1. *Toxicon* **2018**, *143*, 81–89.
129. Genuário, D.; Silva-Stenico, M.; Welker, M.; Moraes, L.; Fiore, M. Characterization of microcystin and detection of microcystin synthetase genes from a Brazilian isolate of *Nostoc*. *Toxicon* **2010**, *55*, 846–854.
130. Sivonen, K.; Namikoshi, M.; Evans, W.; Färdig, M.; Carmichael, W.; Rinehart, K. Three new microcystins, cyclic heptapeptide hepatotoxins, from *Nostoc* sp. strain 152. *Chem. Res. Toxicol.* **1992**, *5*, 464–469.
131. Nagatsu, A.; Kajitani, H.; Sakakibara, J. Muscoride A: A new oxazole peptide alkaloid from freshwater cyanobacterium *Nostoc muscorum*. *Tetrahedron Lett.* **1995**, *36*, 4097–4100.
132. Gehringer, M.; Pengelly, J.; Cuddy, W.; Fieker, C.; Forster, P.; Neilan, B. Host selection of symbiotic cyanobacteria in 31 species of the Australian cycad genus: *Macrozamia* (Zamiaceae). *Mol. Plant. Microbe Interact.* **2010**, *23*, 811–822.

133. Todorova, A.; Jüttner, F. Nostocyclamide: A new macrocyclic, thiazole-containing allelochemical from *Nostoc* sp. 31 (cyanobacteria). *J. Org. Chem.* **1995**, *60*, 7891–7895.
134. Jüttner, F.; Todorova, A.; Walch, N.; von Philipsborn, W. Nostocyclamide M: A cyanobacterial cyclic peptide with allelopathic activity from *Nostoc* sp. 31. *Phytochem.* **2001**, *57*, 613–619.
135. Banker, R.; Carmeli, S. Tenuecyclamide A-D, cyclic hexapeptides from the cyanobacterium *Nostoc spongiaeforme* var. *tenue*. *J. Nat. Prod.* **1998**, *61*, 1248–1251.
